# Real-life data of hypoglycemic events in children and adolescents with type 1 diabetes

**DOI:** 10.1136/bmjdrc-2023-003485

**Published:** 2023-09-22

**Authors:** Henrik Hill, Per Klaar, Daniel Espes

**Affiliations:** 1 Department of Women's and Children's Health, Uppsala University, Uppsala, Sweden; 2 OneTwo Analytics AB, Solna, Sweden; 3 Science for Life Laboratory, Department of Medical Sciences, Uppsala University, Uppsala, Sweden; 4 Science for Life Laboratory, Department of Medical Cell Biology, Uppsala University, Uppsala, Sweden

**Keywords:** continuous glucose monitoring, diabetes mellitus, type 1, pediatrics, hypoglycemia

## Abstract

**Introduction:**

Hypoglycemia composes an always present risk in the treatment of type 1 diabetes (T1D) and can be a fatal complication. Many studies on hypoglycemic events are based on self-reported data or focused on the aggregated time below range. We have processed continuous glucose monitoring (CGM) data in children and adolescents with T1D in order to examine all occurring hypoglycemic events.

**Research design and methods:**

CGM data (mean 168±3 days) from 214 children and adolescents with T1D were analyzed using computer-based algorithms. Patients were divided into three groups based on estimated HbA1c (eHbA1c): (1) ≤48 mmol/mol (n=58); (2) 49–64 mmol/mol (n=113); (3) ≥65 mmol/mol (n=43). The groups were compared concerning descriptive data and CGM metrics with emphasis on the frequency of hypoglycemic events.

**Results:**

Only one self-reported event of severe hypoglycemia was registered, while 54 390 hypoglycemic events (<3.9 mmol/L (<70 mg/dL)) were identified from CGM data out of which 11 740 were serious (<3.0 mmol/L (<54 mg/dL)). On average there were 1.5±0.1 hypoglycemic events per 24 hours out of which 1.2±0.1 were mild (3.0–3.9 mmol/L) and 0.3±0.02 serious. Group 1 had a higher frequency of both total and mild hypoglycemic events compared with both groups 2 and 3. However, the frequency of serious hypoglycemic events was similar in all groups. A negative correlation was observed for eHbA1c and total daily and mild hypoglycemic events (r=−0.57 and r=−0.66, respectively, p<0.0001), whereas for serious hypoglycemic events there was only a borderline significance (r=−0.13, p=0.05).

**Conclusions:**

This study shows that hypoglycemic events are a frequent phenomenon in children and adolescents with T1D, occurring regardless of overall metabolic control. Although patients with an HbA1c ≤48 mmol/mol had a higher frequency of mild hypoglycemic events there was no increase in serious hypoglycemic events.

**WHAT IS ALREADY KNOWN ON THIS TOPIC**

- Since the introduction of continuous glucose monitoring (CGM), rates of severe hypoglycemic events in type 1 diabetes have declined and new ways of defining hypoglycemic events have arisen. Most studies nowadays are either based on self-reported events of severe hypoglycemia or as the aggregated time below range (TBR, <3.9 mmol/L). The frequency of non-severe hypoglycemic events in a modern treatment setting is however not well studied.

**WHAT THIS STUDY ADDS**

- In a real-life setting from long-term registrations of CGM data we found that non-severe hypoglycemic events are a frequent phenomenon in children and adolescents with type 1 diabetes which, on average, occur on a daily basis. However, an HbA1c ≤48 mmol/mol can be achieved without a higher frequency of serious hypoglycemic events (ie, <3.0 mmol/L).

**HOW THIS STUDY MIGHT AFFECT RESEARCH, PRACTICE OR POLICY**

- Including the frequency of hypoglycemic events in addition to the aggregated measure of TBR can contribute to a more detailed and representative description of individual patterns of hypoglycemia which is of importance for a more individualized management approach.

## Introduction

Type 1 diabetes (T1D) is characterized by an immune-mediated destruction of the insulin producing beta cells.^1^ It is one of the most common chronic diseases of childhood and the prevalence is still increasing.^2 3^ In order to reduce the risk of long-term complications it is of utmost importance to maintain glucose levels as close to the normal range as possible. However, this is a balancing act since overestimated insulin doses instead lead to hypoglycemia. Glycosylated hemoglobin (ie, HbA1c) is used as a surrogate marker for long-term (approximately 12 weeks) mean glucose levels and has been widely studied in terms of the risk for complications.^4–6^ However, HbA1c does not take into account glucose fluctuations and an improved HbA1c could mask a high number of hypoglycemic events. In fact, older studies have found that lowering HbA1c increases the risk of severe hypoglycemic events.^4 7 8^ However, with more modern treatment regimes, a low HbA1c is no longer a strong predictor of severe hypoglycemia in T1D.^9–11^ Based on that, guidelines for pediatric T1D management of today in both Sweden and the UK recommend a target HbA1c ≤48 mmol/mol (≤6.5%), although with reservation for the occurrence of severe hypoglycemia or frequent mild hypoglycemic events.^12 13^ Nevertheless, hypoglycemia remains the most common acute complication of T1D. It can give rise to a wide range of symptoms, from mild events with palpitations, sweatiness and shakiness to severe with neurological symptoms, seizures with coma and potentially death.^14 15^ Even mild hypoglycemic events, and also fear of hypoglycemia, are a hurdle for achieving optimal glycemic levels. In addition, hypoglycemia has a negative impact on quality of life for both the affected patients and their families.^16^ Having suffered from a severe hypoglycemic event has also been shown to elevate HbA1c levels, most likely due to safety behaviors, which increases the risk for long-term complications.^17^

In a large number of previous studies, the frequency and occurrence of hypoglycemia have been based on self-reported severe hypoglycemic events.^7 18–20^ Although of great importance, this also comes with several limitations including both the recall bias and the fact that younger children always need aid in resolving the hypoglycemia, leaving it to the parent’s interpretation to assess whether it was by definition a severe event or not. Since the introduction of continuous glucose monitoring (CGM) devices we now have access to an enormous amount of glucose data which makes it possible to identify self-reported hypoglycemic events and the everyday occurrence of both mild and serious hypoglycemic events. This has also led to a modification of the hypoglycemia definition which is now aligned with the cut-off targets for the ambulatory glucose report metrics for CGM data (<3.9 mmol/L) instead of the previous definition of <3.5 mmol/L which was based on the presence of a physiological counter-regulatory hormonal response.^21 22^ According to International Society for Pediatric and Adolescent Diabetes (ISPAD) Clinical Practice Consensus Guidelines, hypoglycemic events can be divided into three groups: (1) clinical hypoglycemia alert (<3.9 mmol/L (<70 mg/dL)), (2) clinically important or serious hypoglycemia (<3.0 mmol/L (<54 mg/dL)) and (3) severe hypoglycemia, that is, events associated with severe cognitive impairment including coma and convulsions requiring external assistance by another person.^23^ In current practice and most research studies, hypoglycemic events are however presented as a percentage of the time spent below range (TBR) (ie, <3.9 and <3.0 mmol/L) rather than the number of specific events of hypoglycemia. In the current study, we have focused on the frequency of hypoglycemic events and the relationship with overall metabolic control in a real-world setting based on retrospective long-time registrations of CGM data in children and adolescents with T1D.

## Research design and methods

### Participants

Data from the time period of 2018–2021 were collected from n=214 patients with T1D followed at Uppsala University Children’s Hospital. The diagnosis of T1D was established according to clinical routine in Sweden, that is, following guidelines from the Swedish Society for Pediatric Endocrinology and Diabetes. Descriptive clinical data were collected from electronic medical records and the Swedish National Diabetes Register. Patients were included based on (1) established diagnosis of T1D, (2) data availability and (3) age ≤19 years as the only inclusion/exclusion criteria. Hence, treatment modality, clinical descriptive or CGM data characteristics were not known in advance. Of the n=214 patients, n=144 (67%) used continuous subcutaneous insulin infusion (CSII), whereas n=70 (33%) used multiple daily injections (MDI). Of the patients with CSII only n=12 (8%) had sensor augmented pump (SAP) therapy with predictive low glucose management (PLGM) algorithms. The included patients were divided into three groups based on estimated HbA1c (eHbA1c) levels; group 1: ≤48 mmol/mol (≤6.5%) (n=58), group 2: 49–64 mmol/mol (6.6–8%) (n=113) and group 3: ≥65 mmol/mol (≥8.1%) (n=43). The cut-off limits of HbA1c were chosen due to the Swedish pediatric guidelines of HbA1c, where the treatment target is ≤48 mmol/mol and an intensified treatment regime and follow-up is recommended for patients with an HbA1c ≥65 mmol/mol because of the increase in long-term complications beyond that level.^4^ The groups were compared concerning descriptive patient data and CGM-derived metrics with emphasis on the occurrence and frequency of hypoglycemic events. For full descriptive data, see table 1.

**Table 1:** Descriptive data and ambulatory glucose profile metrics of study participants

Parameter	Group 1 eHbA1c ≤48 mmol/mol	Group 2 eHbA1c 49–64 mmol/mol	Group 3 eHbA1c ≥65 mmol/mol
Total, n	58	113	43
Female, n (%)	27 (45.0)	55 (48.7)	20 (48.8)
Age (years)	12.6±0.5	12.7±0.4	15.0±0.5**
Duration (years)	4.2±0.5	5.4±0.3	6.2±0.5**
Insulin (IU/kg/24 hours)	0.66±0.02	0.73±0.02	0.84±0.05****
BMI (kg/m^2^)	20.5±0.4	20.8±0.4	22.7±0.8*
Overweight and obese (iso-BMI), n (%)	15 (25.9)	37 (32.7)	16 (37.2)
Celiac disease, n (%)	7 (12.1)	11 (9.7)	4 (9.3)
Hypothyreosis, n (%)	3 (5.2)	4 (3.5)	5 (11.6)
Continuous subcutaneous insulin infusion, n (%)	40 (69.0)	79 (69.9)	25 (58.1)
Multiple daily injections, n (%)	18 (31.0)	34 (30.1)	18 (41.9)
rtCGM, n (%)	30 (51.7)	73 (64.6)**	14 (32.6)**
isCGM, n (%)	28 (48.3)	40 (35.4)**	29 (67.4)**
Days with CGM readings	165.3±5.9	171.6±4.0	161.7±6.3
eHbA1c (mmol/mol)	43.2±0.5	55.5±0.4****	73.0±1.3****
Mean glucose (mmol/L)	7.2±0.1	9.0±0.1****	11.5±0.2****
Time in range (%)	75.3±1.4	59.8±0.7****	39.2±1.1****
Time in tight range (%)	56.0±1.3	38.5±0.6****	23.4±0.8****
Time below range (%)	9.0±0.8	5.7±0.4***	5.2±0.6**
Time above range (%)	15.7±0.8	34.5±0.6****	55.6±1.2****
Coefficient of variation (CV%)	39.5±1.0	42.4±0.5*	45.3±0.9****
SD (mmol/L)	2.9±0.1	3.8±0.1****	5.2±0.1****

Study participants were grouped based on their eHbA1c from continuous glucose monitoring (CGM) data from the full time period of available data. All other CGM-derived metrics were also calculated for the full time period of available data. Statistical comparisons were performed with a one-way ANOVA (Kruskal-Wallis) using Dunnett’s multiple comparison post hoc test for comparison with group 1. Non-numeric parameters were compared using a χ^2^ test. All applicable values are given as mean±SEM. P values <0.05 were considered statistically significant.

*P<0.05, **p<0.01, ***p<0.001, ****p<0.0001 when compared with group 1.

ANOVA, analysis of variance; BMI, body mass index; CGM, continuous glucose monitoring; eHbA1c, estimated HbA1c (based on CGM data from the full time period of available data); isCGM, intermittent-scanning CGM; rtCGM, real-time CGM.

### Data preparation

The retrospective CGM data were downsampled to 15 min intervals in order to harmonize the data between different CGM models, that is, intermittent-scanning CGM (isCGM) and real-time CGM (rtCGM). CGM-derived metrics were computed for the full extent of available data for each patient (range 29–200 days, mean 168±3 days). Standard metrics computed include mean glucose, SD, coefficient of variation (CV%), eHbA1c (calculated using the formula: mmol/mol=average glucose (mmol/L)×6.94–6.63), % of TBR (<3.9 mmol/L), % of time spent in tight range (TITR, 3.9–7.8 mmol/L), % of time spent in range (TIR, 3.9–10.0 mmol/L) and % of time spent above range (TAR, ≥10.1 mmol/L). In addition, all unique events of hypoglycemia were identified. An event was defined as each episode with a glucose level <3.9 mmol/L lasting until the glucose levels returned to ≥3.9 mmol/L. The event was classified as serious if the lowest value during the event was <3.0 mmol/L. For each event, duration, lowest glucose level and time of day were registered. The number of events was normalized for days with CGM readings (ie, events/24 hours) in order to make it possible to compare the frequency of hypoglycemia. Events occurring between 22:00 and 06:00 were defined as nightly hypoglycemic events. The CGM data were processed by in-house computer-based algorithms developed by OneTwo Analytics (Solna, Sweden). Descriptive clinical data were collected from electronic medical records. The data were primarily from patient visits during the period of collected CGM data but if no visit was registered during that time period, data from the visit closest in time to that period were registered instead. The collected data included age, gender, weight, height, body mass index (BMI), iso-BMI, disease duration, occurrence of celiac disease and hypothyroidism, insulin treatment modality (MDI or CSII), insulin doses (IU/kg/day) and self-reported events of severe hypoglycemia.

### Statistical methods

Statistical analyses were conducted with GraphPad Prism V.9.3.0. Comparison between three groups was performed with a one-way analysis of variance (Kruskal-Wallis) using Dunnett’s multiple comparison post hoc test for comparison with group 1. Non-numeric parameters were compared using χ^2^ test when comparing three groups and Fisher’s exact test when comparing two groups. Comparisons between two groups (ie, MDI vs CSII) were performed using a Student’s t-test for parameters that passed the D’Agostino and Pearson normality test or a Mann-Whitney test for non-normality distributed data. Correlations were computed with Spearman rank-order test. Data are presented as means±SEM. P values <0.05 were considered statistically significant.

## Results

### Descriptive data

The patients in group 1 (eHbA1c ≤48 mmol/mol) were younger (12.6±0.5 vs 15.0±0.5 years, p<0.01) and had a shorter mean duration of T1D (4.2±0.5 vs 6.2±0.5 years, p<0.01) when compared with the patients in group 3 (eHbA1c ≥65 mmol/mol). Also, the patients in group 1 had lower daily insulin doses (0.66±0.02 vs 0.84±0.05 IU/kg/24 hours, p<0.0001) compared with group 3. Group 1 was less frequently equipped with rtCGM versus isCGM than group 2, but more frequently than group 3 (p<0.01, respectively). We observed no differences for gender, obesity, occurrence of celiac disease or hypothyroidism, nor in treatment modality (MDI vs CSII) or days with CGM readings. The full descriptive data are presented in table 1.

### Overall glucose control

As by design, group 1 had a lower eHbA1c and in line with that a lower mean glucose and a higher percentage of TIR and TITR (table 1). Interestingly, the TBR was also higher in group 1 compared with both groups 2 and 3 (9.0±0.8 vs 5.7±0.4 and 5.2±0.6%, respectively, p<0.01). All CGM-derived metrics are presented in table 1.

### Hypoglycemic events

**Figure 1:**
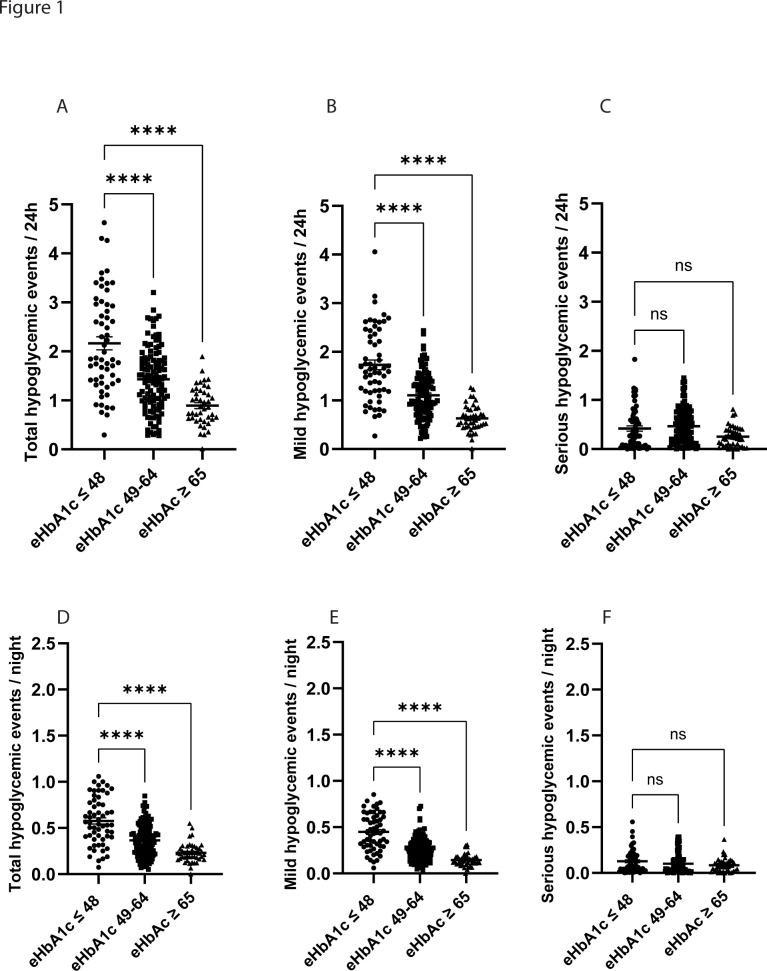
Frequency of hypoglycemic events in children and adolescents with type 1 diabetes. Patients with type 1 diabetes who reach the current treatment target of an HbA1c ≤48 mmol/mol estimated based on long-term readings of continuous glucose monitoring (CGM) data (estimated HbA1c, eHbA1c) have a higher frequency of both total and mild (3.0–3.9 mmol/L) hypoglycemic events (A, B) but not serious (<3.0 mmol/L) hypoglycemic events (C). The same pattern was observed when comparing only hypoglycemic events occurring at night (22:00–06:00), that is, the patients reaching the target of eHbA1c ≤48 mmol/mol had a higher frequency of total and mild hypoglycemic events (D, E) but not serious hypoglycemic events (F). The total cohort of n=214 patients was divided into three groups based on eHbA1c: eHbA1c ≤48 mmol/mol (n=58), eHbA1c 49–64 mmol/mol (n=113) and eHbA1c ≥65 mmol/mol (n=43). Values are presented as individual values and mean±SEM is indicated in the figure. ****P<0.0001.

**Figure 2:**
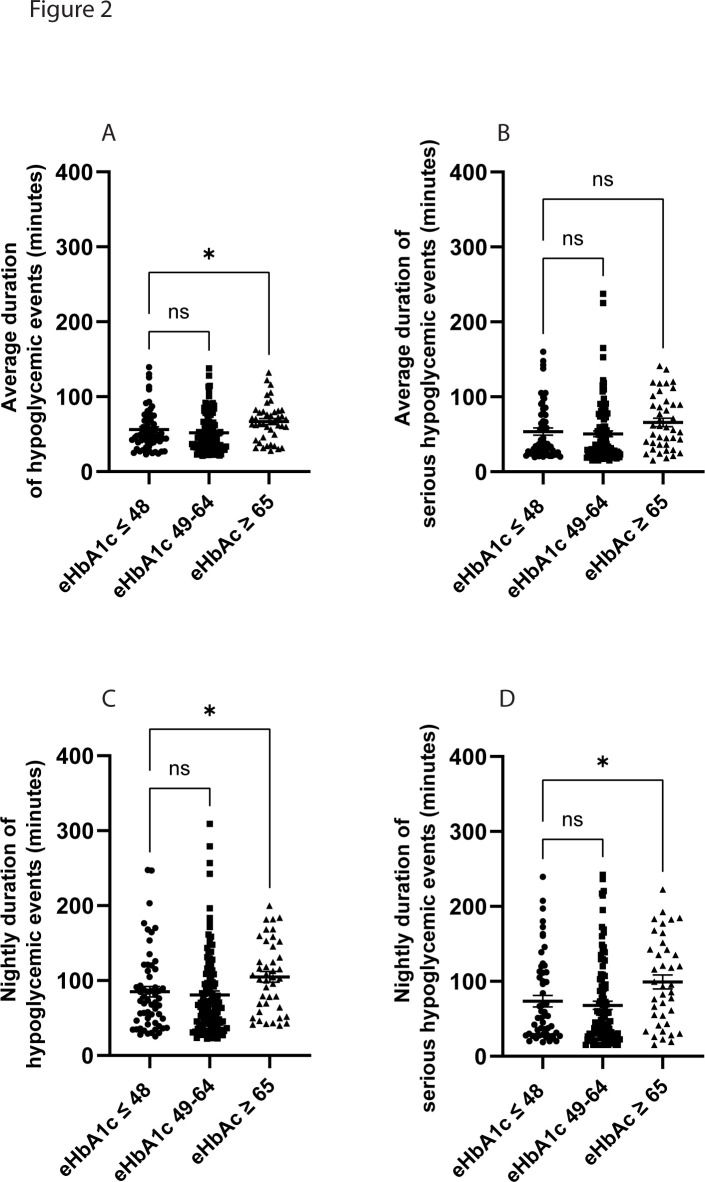
Duration of hypoglycemia. The average duration (min) of overall hypoglycemic events and serious hypoglycemic events was calculated for each participant. The patients with the highest mean estimated HbA1c (eHbA1c) levels (≥65 mmol/mol) had a longer average duration of hypoglycemia when compared with the patients with the lowest mean eHbA1c (≤48 mmol/mol) (A) but not for serious hypoglycemic events (<3.0 mmol/L) (B). During night-time (22:00–06:00) the patients with the highest mean eHbA1c had a longer duration for both overall hypoglycemic events (C) and serious hypoglycemic events (D) when compared with the patients with the lowest mean eHbA1c. The total cohort of n=214 patients was divided into three groups based on eHbA1c: eHbA1c ≤48 mmol/mol (n=58), eHbA1c 49–64 mmol/mol (n=113) and eHbA1c ≥65 mmol/mol (n=43). Values are presented as individual values and mean±SEM is indicated in the figure. *P<0.05.

Noteworthy, for the whole time period there was only one self-reported event of severe hypoglycemia (group 2). This is in great contrast with the 54 390 total hypoglycemic events identified by CGM. Of the total hypoglycemic events, 11 740 (21%) were serious (ie, <3.0 mmol/L). The overall mean for total hypoglycemic events was 1.5±0.1 events/24 hours out of which 1.2±0.1 were mild and 0.3±0.02 were serious events. In other words, hypoglycemic events were common and occurred on average on a daily basis. Group 1 had a higher frequency of total and mild hypoglycemic events when compared with groups 2 and 3. However, the frequency of serious hypoglycemic events was similar in all groups (figure 1). The same pattern was identified for nightly hypoglycemic events (figure 1). Interestingly, group 1 had a shorter duration of overall hypoglycemic events during both day and night and also for serious hypoglycemic events during the night when compared with group 3 (figure 2).

### Correlations of hypoglycemic events and overall glucose control

**Figure 3:**
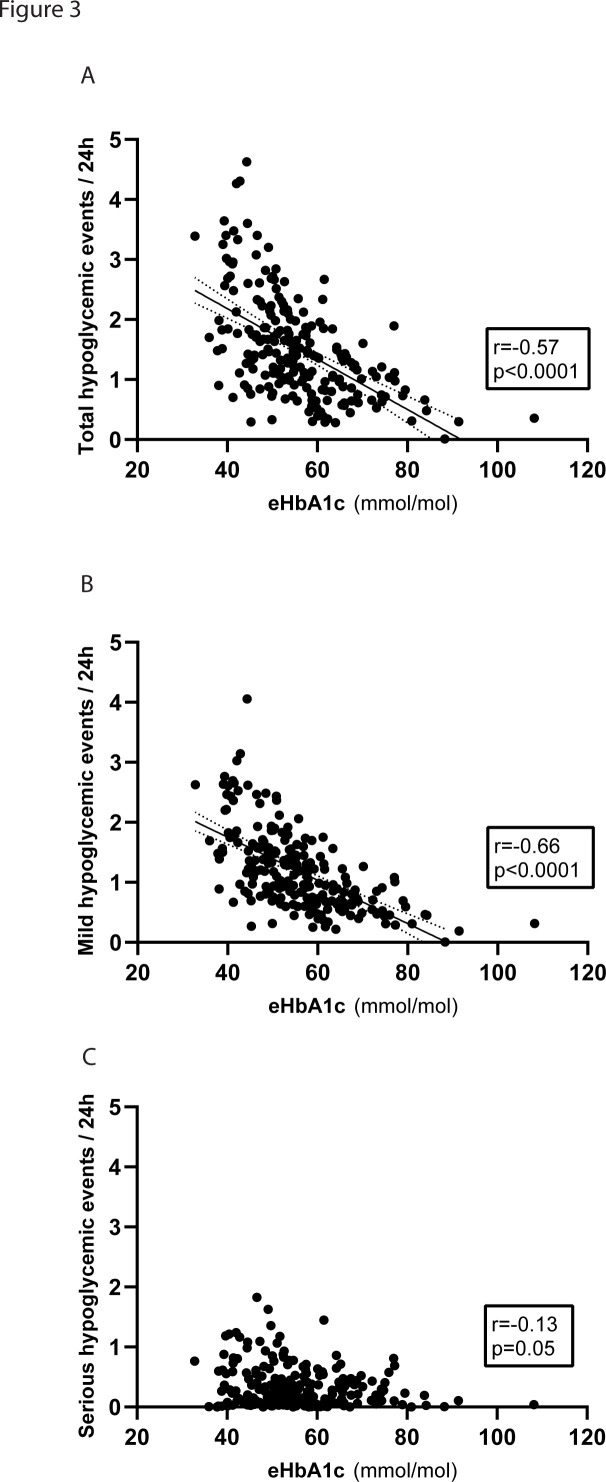
Correlation of the frequency of hypoglycemic events with estimated HbA1c (eHbA1c). Correlation was computed based on eHbA1c from continuous glucose monitoring (CGM) readings and the frequency of hypoglycemia for the whole cohort of n=214 children and adolescents with type 1 diabetes. A negative correlation between eHbA1c and the total daily hypoglycemic events (A) and mild hypoglycemic events (B) was observed. For serious hypoglycemic events there was a borderline significant correlation with eHbA1c (C). Correlations were computed using a Spearman rank-order test. The lines in (A) and (B) represent a simple linear regression and the dotted lines the 95% confidence bands.

We observed a negative correlation between eHbA1c and total daily and mild hypoglycemic events (r=−0.57 and r=−0.66, respectively, p<0.0001), while for serious hypoglycemic events there was a borderline significant correlation (r=−0.13, p=0.05) (figure 3). As expected, a positive correlation was observed between TBR and frequency of hypoglycemic events (online supplemental table 1). In line with the correlations of eHbA1c, TIR also correlated positively with both the total number of hypoglycemic events and mild hypoglycemic events (r=0.32 and r=0.51, respectively, p<0.0001). Interestingly, for serious hypoglycemic events we instead observed a negative correlation with TIR (r=−0.17, p=0.015). TAR correlated negatively with both the total number of hypoglycemic events and mild hypoglycemic events (r=−0.52 and r=−0.63, respectively, p<0.0001), but not with serious hypoglycemic events (r=−0.06, p=0.36). Glucose variability, expressed as CV%, correlated positively with the total number of hypoglycemic events and with serious hypoglycemic events (r=0.37 and r=0.64, respectively, p<0.0001) but not with mild events (r=0.13, p=0.06). For SD of glucose on the other hand, a negative correlation was observed with the total number of hypoglycemic events (r=−0.17, p=0.012) and mild hypoglycemic events (r=−0.37, p<0.0001) but not for serious hypoglycemic events; as for CV%, a positive correlation was observed (r=0.26, p<0.0001).

10.1136/bmjdrc-2023-003485.supp1 Supplementary data

When correlating hypoglycemic events with descriptive clinical parameters we found that age correlated negatively with both the total number of hypoglycemic events and mild hypoglycemic events (r=−0.33 and r=−0.44, respectively, p<0.0001) but not with serious events (r=−0.05, p=0.45). Interestingly, disease duration correlated negatively with mild hypoglycemic events (r=−0.15, p=0.029) but positively with serious events (r=0.23, p<0.001). We observed no correlations between hypoglycemic events and daily insulin requirements. All computed correlations are presented in online supplemental table 1.

### Hypoglycemic events in patients with MDI versus CSII

Patients using MDI were slightly older (14.1±0.4 vs 12.6±0.3 years, p<0.01), had a shorter duration of T1D (4.5±0.4 vs 5.6±0.3 years, p<0.01) and a higher presence of obesity compared with patients using CSII (40.1% vs 26.6%, p=0.041). We found no differences between the two treatment modalities concerning eHbA1c, TIR, TBR or any of the other ambulatory glucose profile parameters. Neither were there any differences in the frequency of hypoglycemic events; however, there was a tendency toward a slight increase in mild hypoglycemic events for patients using CSII (1.25±0.06 vs 1.07±0.07 events/24 hours, p=0.052). Patients with MDI had a longer average duration of hypoglycemia (61.9±3.0 vs 53.2±2.2 min, p<0.01) as well as for serious hypoglycemic events (64.9±4.2 vs 49.4±3.3 min, p<0.001). This was true also when analyzing the duration of nightly hypoglycemic events (96.4±6.4 vs 82.0±4.4 min, p=0.021) as well as nightly serious hypoglycemic events (91.0±6.9 vs 68.0±4.9 min, p<0.01). In the data set there were only n=12 of the patients with CSII who had PLGM, but in these individuals we observed a shorter duration of hypoglycemia when compared with the remaining patients using insulin pump (n=132) (35.3±3.1 vs 54.5±2.4 min, p<0.01). This cannot, however, explain the difference when compared with patients using MDI since the duration of hypoglycemia was longer in this group even when compared with the patients using CSII after excluding those with PLGM (62.5±3.0 vs 54.5±2.4 min, p=0.043). All the descriptive and CGM-derived data for the comparison of patients with MDI versus CSII are presented in table 2.

**Table 2:** Comparison of descriptive data and CGM-derived metrics between patients with multiple daily injections and continuous subcutaneous insulin infusion

Parameter	MDI	CSII
Total, n	70	144
Female, n (%)	35 (50.0)	67 (46.5)
Age (years)	14.1±0.4	12.6±0.3**
Duration (years)	4.5±0.4	5.6±0.3**
BMI (kg/m^2^)	22.2±0.5	20.56±0.3**
Overweight/obese (iso-BMI), n (%)	29 (40.1)	38 (26.6)*
Insulin needs (IU/kg/24 hours)	0.76±0.03	0.72±0.02
Days with CGM	167.8±4.7	167.9±3.7
eHbA1c (mmol/mol)	57.1±1.6	55.0±0.9
Mean glucose (mmol/L)	9.2±0.2	8.9±0.1
Glucose SD (mmol/L)	4.0±0.1	3.7±0.1
Coefficient of variation (CV%)	42.6±0.9	42.0±0.5
Time below range (%)	6.6±0.5	6.4±0.4
Time in range (%)	58.2±2.1	60.7±1.1
Time above range (%)	35.1±2.1	32.9±1.2
Time in tight range (%)	39.5±1.9	40.5±1.0
Average of total hypoglycemic events per day	1.39±0.09	1.59±0.07
Average of mild hypoglycemic events per day	1.07±0.07	1.25±0.06
Average of serious hypoglycemic events per day	0.32±0.04	0.34±0.03
% of hypoglycemic events that were serious	21.5±2.2	19.2±1.2
Nightly average of hypoglycemic events	0.36±0.03	0.41±0.02
Nightly average of serious hypoglycemic events	0.10±0.01	0.11±0.01
% of hypoglycemic events occurring at night	27.2±1.1	26.9±0.7
Average hypoglycemia duration (min)	61.9±3.0	53.2±2.2**
Average nightly hypoglycemia duration (min)	96.4±6.4	82.0±4.4*
Average duration of serious hypoglycemic events (min)	64.9±4.2	49.4±3.3***
Average nightly duration of serious hypoglycemic events (min)	91.0±6.9	68.0±4.9**
Hypoglycemic events >60 min (%)	33.7±2.0	27.2±1.5**
Serious hypoglycemic events >60 min (%)	66.6±3.4	53.9±2.3**

Coefficient of variation, time in range, time in target and time above range passed the D’Agostino and Pearson normality distribution test and were compared using a Student’s t-test. Distribution of iso-BMI as overweight/obese was compared with Fisher’s exact test. All other parameters were compared using a Mann-Whitney test. All applicable values are presented as mean±SEM. P values <0.05 were considered statistically significant.

*P<0.05, **p<0.01 and ***p<0.001.

BMI, body mass index; CGM, continuous glucose monitoring; CSII, continuous subcutaneous insulin infusion; eHbA1c, estimated HbA1c; MDI, multiple daily injections.

## Discussion

Our real-life CGM data clearly show that hypoglycemia is a frequent phenomenon among children and adolescents with T1D. Of great interest, a total of 11 740 serious hypoglycemic events (<3.0 mmol/L) were registered in the data set while only one severe hypoglycemic event was reported during the same time period. There could be a number of reasons for this great discrepancy and it is important to keep in mind that events classified as serious based on the numeric glucose level do not reveal whether or not it caused any symptom or required assistance which a severe event does by definition. Regardless, this great numerical difference between serious and severe hypoglycemic events highlights the limitations of relying on self-reported hypoglycemic episodes. Interestingly, we found that the number of serious hypoglycemic events was positively correlated with disease duration, that is, the number of serious hypoglycemic events increases over time. This is in line with a recent observation of ours based on reported severe hypoglycemic events which is more common in pediatric patients with a rapid decline of endogenous C peptide.^24^ In the current cohort, C peptide data were not available, but it would be of great interest to study how the frequency of serious hypoglycemic events evolves in relation to the progressive loss of endogenous insulin production.

The rates of severe hypoglycemia in children and adolescents have been found to decrease in developed countries during the last decades and a low HbA1c is no longer associated with an increased rate of severe hypoglycemia thanks to modern treatment approaches.^9–11^ This was a part of the argumentation for lowering the target for HbA1c to ≤48 mmol/mol, as implemented in Sweden and the UK.^12 13^ In this study, we used eHbA1c based on long CGM registrations to compare the groups, which previously has been proven to correlate well to laboratory-based measurements.^25^ We found that even though there was an increase in both the total number of hypoglycemic events and mild hypoglycemic events, there was no increase in the occurrence of serious hypoglycemic events in patients with an eHbA1c ≤48 mmol/mol. In line with that, both the total number of hypoglycemic events and mild hypoglycemic events were negatively correlated with eHbA1c, whereas for serious hypoglycemic events there was only a borderline significant correlation with eHbA1c (r=−0.13, p=0.05). However, a negative correlation between serious hypoglycemic events and TIR was observed which also supports the notion that an overall improved glycemic control is not associated with more frequent serious hypoglycemic events. Also, in the group with an eHbA1c ≤48 mmol/mol, the overall duration of hypoglycemic events during both day and night was shorter when compared with the group with the highest eHbA1c (≥65 mmol/mol). During the night, this was also true for serious hypoglycemic events.

According to ISPAD Clinical Practice Consensus Guidelines of 2022,^23^ TBR should be less than 4%. In our study based on real-life data none of the groups meet this criterion. In fact, those who reached the treatment target for HbA1c had an average TBR twice as high as the guidelines stipulate (9.0±0.8%, corresponding to 2.2 hours daily) and more frequent mild hypoglycemic events when compared with the two other groups. It is important to highlight that the national guidelines in the UK and Sweden take into account the frequency of hypoglycemic events, an aspect that is not accounted for in the current standard of reporting CGM data despite that this is part of the guidelines used in clinical practice. Also, to the best of our knowledge, there is no clear and accepted definition of what would be considered as frequent episodes. Hence, this is a current challenge from a clinical perspective when adopting a stricter HbA1c target since we currently are not adequately monitoring the frequency of mild or serious hypoglycemic events. This has a major impact from a patient perspective, since the lived experience of frequently occurring hypoglycemic events during daily activities is dramatically different compared with one longer episode of a glucose level of 3.8 mmol/L during the night. However, the two different scenarios could theoretically give rise to the same percentage of TBR. In addition, over time hypoglycemic events may also contribute to long-term complications. Although the underlying mechanisms have not yet been fully depicted there are publications which imply that hypoglycemia is a risk factor for long-term cognitive complications.^26 27^ Hence, in addition to the aspect of quality of life it is of importance to monitor and strive to minimize the number of hypoglycemic events.

In Sweden, CGM has become standard of care and only a small number of pediatric patients still use self-monitoring of blood glucose (SMBG). Our data contain glucose data derived from both isCGM, without predictive alerts for hypoglycemic events, and rtCGM with such alerts. It has previously been shown that both of these systems have the potential to reduce hypoglycemia,^28 29^ although the reduction is greater in rtCGM with alert functionality.^30^ In a setting where SMBG is still the standard of care, TBR could therefore be expected to be even further from the target of 4%.

Mild hypoglycemic events correlated positively with TIR and negatively to TAR, as opposed to serious events which correlated negatively with TIR but were uncorrelated to TAR. Also, serious hypoglycemic events were positively correlated with both CV% and SD of glucose indicating that patients with serious hypoglycemic events have a more fluctuating glucose, where time spent in desired glucose range is shifted toward TBR, while TAR unfortunately is unaffected. This is in line with a previous study where glucose variability independently predicted time spent in hypoglycemia.^31^

Concerning insulin treatment modality, the group equipped with CSII was of younger age and had a longer T1D duration than the MDI group. This can probably be attributed to the current clinical approach in which MDI is most often the default modality initiated after diagnosis and a more active approach toward equipping children of younger age with insulin pumps after debut. In contrast to previous studies, which have shown a lower HbA1c in patients on CSII compared with those treated with MDI,^32 33^ we observed no difference in eHbA1c. However, the group with CSII had a shorter duration of hypoglycemia compared with the MDI group, but no differences were observed for other CGM metrics. During the end of the time period for data collection, SAP therapy with PLGM algorithms entered the market, but only n=12 (8%) of the patients with CSII were treated with such insulin pumps. PLGM can suspend insulin therapy when hypoglycemia is either predicted or present, and the use of PLGM has been shown to reduce time spent in hypoglycemia compared with SAP therapy alone.^34 35^ Such insulin pumps could hence improve the chance of reaching the combined treatment goal of an HbA1c ≤48 mmol/mol without frequent hypoglycemic events. In addition, more recently pumps with automated insulin delivery (AID) systems, also known as closed loop, have been introduced in the clinical setting, which could further enhance the likelihood of reaching the glycemic targets while mitigating the frequency of hypoglycemia.^36–39^

### Strengths and limitations

CGM data from extended time periods in a real-life setting in combination with clinical descriptive data from electronic patient records are the key strengths of this study. The computation of the number of hypoglycemic events in addition to TBR is also an advantage since the discrepancy between the two could have major implications for the lived experience of hypoglycemia.

In the harmonization of data from rtCGM and isCGM, by downsampling readings to 4 per hour, hypoglycemic events could theoretically have been missed which would underscore the frequency of hypoglycemia. On the other hand, the duration of short episodes of hypoglycemic events could have been overestimated.

Due to the retrospective nature of the study, we lack data regarding confounding factors to hypoglycemia (such as physical activity, sleep pattern, etc) which is a limitation. In addition, we do not have any data regarding symptoms of hypoglycemia and/or quality of life in relation to the frequency of hypoglycemia. There could also be a number of false hypoglycemic events (ie, compression lows) which could especially impact the number and duration of hypoglycemic events during the night. In addition, the data set contains glucose values from different types of sensors which introduces both differences in regard of accuracy but also implications for the everyday life management of hypoglycemia pending on whether or not the sensor had an alarm function. Also, the data were collected at a single center and it would be of interest to verify these findings on a national level, but also to compare with data from other countries in which the approach to management of hypoglycemia may differ. Future prospective studies collecting CGM data of hypoglycemic events coupled with registration of symptoms, confounding factors, treatment of hypoglycemia and data concerning quality of life are highly needed and would be of great clinical importance.

## Conclusion

This single-center study shows that hypoglycemic events are a frequent phenomenon in children and adolescents with T1D, occurring regardless of overall metabolic control. Although patients with an HbA1c ≤48 mmol/mol have a higher frequency of mild hypoglycemic events, there was no increase in serious hypoglycemic events. In other words, we found that in a real-world setting the treatment targets regarding HbA1c can be achieved without an increased frequency of either serious or severe hypoglycemic events, but not without an elevation of mild hypoglycemic events. Also, the hypoglycemic target of a TBR <4% set by ISPAD Clinical Practice Consensus Guidelines of 2022^23^ was not met in any of our examined groups. Future studies of real-world CGM data are needed to elaborate if the more recently introduced pumps with AID systems can help close this gap.

## Data Availability

Data are available upon reasonable request. Not applicable.
