# Supplementary material for: Real-life data of hypoglycemic events in children and adolescents with type 1 diabetes
Source: BMJ Open Diabetes Res Care. 2023 Sep 22;11(5):e003485. doi: 10.1136/bmjdrc-2023-003485 (PMC10533671; doi:10.1136/bmjdrc-2023-003485)
Supplement: Supplementary data [file bmjdrc-2023-003485supp001.pdf]

Supplementary Table 1. Correlation of hypoglycemic events with descriptive parameters and CGM-derived metrics

Parameter	Total hypoglycemic events / 24h		Mild hypoglycemic events / 24h		Serious hypoglycemic events / 24h	
	r	p	r	p	r	p
eHbA1c	-0.57	<0.0001****	-0.66	<0.0001****	-0.13	0.050
Glucose level mean	-0.55	<0.0001****	-0.66	<0.0001****	-0.13	<0.050*
% Time below range	0.74	<0.0001****	0.53	<0.0001****	0.77	<0.0001****
% Time in range	0.32	<0.0001****	0.51	<0.0001****	-0.17	0.015*
%Time in target	0.46	<0.0001****	0.63	<0.0001****	-0.04	0.578
% Time above range	-0.52	<0.0001****	-0.63	<0.0001****	-0.06	0.357
Glucose level STD	-0.17	0.012*	-0.37	<0.0001****	0.26	0.0001***
Glucose level CV%	0.37	<0.0001****	0.13	0.063	0.64	<0.0001****
Age	-0.33	<0.0001****	-0.44	<0.0001****	-0.05	0.446
Age at onset	-0.30	<0.0001****	-0.30	<0.0001****	-0.26	<0.001***
Duration of T1D	-0.01	0.843	-0.15	0.029*	0.23	<0.001***
IU/kg/Day	-0.01	0.088	-0.07	0.326	0.11	0.127
BMI	-0.19	<0.01**	-0.27	<0.0001****	-0.02	0.717
Average duration of hypoglycemic events	0.16	0.021*	-0.06	0.404	0.43	<0.0001****
Average duration of serious hypoglycemic events	0.05	0.444	-0.12	0.079	0.30	<0.0001****
Average duration of hypoglycemic events at night	0.17	0.015*	-0.03	0.708	0.40	<0.0001****
Average duration of serious hypoglycemic events at night	0.10	0.146	-0.10	0.159	0.39	<0.0001****
Total hypoglycemic events > 60 minutes	0.70	<0.0001****	0.51	<0.0001****	0.70	<0.0001****
Total serious hypoglycemic events >60 minutes	0.58	<0.0001****	0.29	<0.0001****	0.92	<0.0001****
% total hypoglycemic events >60 minutes	0.19	<0.01**	-0.04	0.586	0.47	<0.0001****
% serious hypoglycemic events >60 minutes	0.03	0.670	-0.09	0.187	0.17	0.015*
Nightly total hypoglycemic events	0.83	<0.0001****	0.77	<0.0001****	0.57	<0.0001****
Nightly mild hypoglycemic events	0.83	<0.0001****	0.77	<0.0001****	0.57	<0.0001****
Nightly serious hypoglycemic events	0.52	<0.0001****	0.30	<0.0001****	0.91	<0.0001****
% of total hypoglycemic events occurring at night	-0.21	<0.01**	-0.20	<0.01**	-0.14	0.042*
% serious hypoglycemic events occurring at night	-0.22	<0.001***	-0.20	<0.01**	-0.23	0.0007***
% serious hypoglycemic events	0.31	<0.0001****	-0.03	0.650	0.87	<0.0001****
Total daily hypoglycemic events	x	x	0.92	<0.0001****	0.68	<0.0001****
Daily mild hypoglycemic events	0.92	<0.0001****	x	x	0.39	<0.0001****

Daily serious hypoglycemic events	0.68	<0.0001****	0.39	<0.001***	X	X

Correlations were computed for the whole study cohort (n=214) using Spearman rank-order test. Mild hypoglycemic events were defined as glucose 3.0 - 3.9 mmol/L and serious hypoglycemic events as < 3.0 mmol/L. Nightly hypoglycemic events were defined as those events occurring between 10PM to 6AM. R-values and p-values are presented for each comparison.
